# Editorial: Examining community-engaged and participatory research programs and projects

**DOI:** 10.3389/fpubh.2023.1239670

**Published:** 2023-07-21

**Authors:** Milton (Mickey) Eder, Karen T. D'Alonzo, Michael A. Yonas, John G. Oetzel

**Affiliations:** ^1^Department of Family Medicine and Community Health, Medical School, University of Minnesota, Minneapolis, MN, United States; ^2^Rutgers Biomedical and Health Sciences, Rutgers, The State University of New Jersey, Newark, NJ, United States; ^3^The Pittsburgh Foundation, Pittsburgh, PA, United States; ^4^School of Management and Marketing, University of Waikato, Hamilton, New Zealand

**Keywords:** community-engaged research, participatory research, community involvement, research implementation, community-based participatory research

The number of community-engaged and participatory research projects has greatly increased over the past few decades, contributing to both additions and refinements in methods and to statistics and stories about research studies and their outcomes. By integrating community expertise and experience, community-engaged and participatory research projects have increased knowledge and applied that knowledge to create social, environmental and political interventions and programs to benefit communities (1, 2). While a clearly recognized canon and discipline has yet to emerge, we have a sufficient foundation for dialogue that is so essential for advancing science (3–6). We assume dialogue will help establish a consistent use of terminology. However, inconsistencies in using terms about community engagement reflect inherent challenges in building evidence from descriptions of local contexts that appear to and that may lack empirical sameness (7, 8). The importance of recognizing local contexts for how we understand and talk about the integration of theory and practice for community engagement in research and for partnership and interpersonal dynamics has been elevated by the attention to middle range theory in Realist Evaluation (9–14). Understanding the local as more than merely a physical space takes on an added significance given the international and national contexts included within this Research Topic. Inconsistencies in the use of terminology is further confounded by the diversity of disciplines, by community histories and personal experiences, and by the different approaches to conducting research and creating knowledge that inform the research and author teams.

We selected the following keywords to indicate the scope of the Research Topic: Community-engaged research, participatory research, community involvement, research implementation. Additionally, and not surprisingly, this Research Topic includes reports that utilize the following terms: Community-based participatory research (Ramji et al.), community-engaged research (Hallmark et al.), both community-based participatory research and community-engaged research (Sanchez-Youngman et al.), community-academic engagement (Chinekezi et al.), community-academic partnership (Chak and Carminati), and participatory health research (Gilfoyle, Salsberg, et al.). Further, the varied forms of and terms for community engagement also allows researchers and community partners to utilize different study designs. The methodologies in this Research Topic include a mixed methods qualitative study (Sanchez-Youngman et al.), a social network analysis (Gilfoyle, Salsberg, et al.), a randomized control trial (Sinclair et al.), a historical or retrospective review (Wieland et al.), a deliberative democracy approach (Guan et al.), and a longitudinal cohort study (Mau et al.). As different as each is one from another, each design reflects an overarching commitment to the science of community engagement. The variation in terminology and differences in research methods challenges scholars internationally to integrate and synthesize not only study designs and methods but also research data.

The essays in this Research Topic are informed by a history of scholarship about engaged and participatory research and their record of achievement in improving community health outcomes. Our editorial team sought to make a unique contribution to the literature by encouraging each manuscript to demonstrate methodological rigor and self-reflectivity. From problem development through outcome assessment, implementation and dissemination, self-reflection in the form of critical consciousness can assume many forms. Our editorial intention to critical reflection in the assessment of context and strategies for overcoming bias is evident in a study that reviews and reflects on discourses about public and patient research engagement within global and national contexts, recommending public and patient leadership of health research and the development of effective partnerships for co-learning that could then drive policy (Gilfoyle, Macfarlane, et al.). Another study, references Leadership Complexity Theory, exploring effective leadership amidst complexities within community-academic partnerships in Germany (Chak and Carminati). Yet another study pursued a nuanced and multidimensional social network analysis for conceptualizing, operationalizing and measuring trust within participatory partnerships (Gilfoyle, Salsberg, et al.). Alternatively, another study advocates academic partners move beyond measuring trust to demonstrate they are trustworthy (Chinekezi et al.). We believe self-reflection informs the study of an extended community's engagement with diabetes across geographically distant locations (Andersen et al.). We encourage you as readers to determine whether the studies in this Research Topic pursue self-reflection along with other forms of commentary and critique.

Studies within this Research Topic specifically highlight the importance of local context. One research team engaged healthcare workers in Belgium in dialogue about pandemic-related issues to study participant expectations and experiences and how social position, role status and power dynamics influenced that dialogue (Nguyen et al.). A comparative case study of physical activity promotion closely examined co-creation of an intervention through cooperative planning and the subsequent transference and implementation of that intervention into three settings (Popp et al.). Comparisons of local contexts and the application of knowledge are at the center of the Family Listening/Family Circle Program with its focus on family communication and connectedness to culture and language; the study authors recommended community action projects in which participants serve as agents for change by developing community solutions based on indigenous values and practices (Rae et al.). The focus on specific local contexts in these reports may appear to accentuate the contrast between the research goal of developing generalizable knowledge and program evaluation with its focus on outcomes within specific localities. This suggested distinction begins to erode with the emergence of discourse around the diffusion of innovation, knowledge transfer, translation, implementation and dissemination, all of which can be understood as middle range theories.

The immediacy of the study topic reported on may itself demonstrate a self-reflective point of departure as is evident in a qualitative research study focused on ways to reduce social isolation and enhance mental and physical wellbeing before and during the pandemic (Ramji et al.). Reflection on the moment appears within work to create a peer-led intervention to promote COVID testing in a public housing community (Plunk et al.). In another study, community academic partnerships utilized the approach of Paulo Freire to develop a study in which the community members become agents of change through their identification and commitment to work on issues of importance to them (Rae et al.). Similarly, a study pondering challenges involved in identifying domains and competencies to inform an educational program included outcomes involving interpersonal skills and partnership development, requiring introspection by trainees in clinical and translational science (Hallmark et al.).

This Research Topic also includes manuscripts that reflect on how institutional policies, practices, and the infrastructures that have emerged to support research implementation might affect partnership and scientific inquiry. For example, institutional and community critiques and challenges were explored through a summary of an 18-year community-based participatory research (CBPR) partnership in Southeast Minnesota (Wieland et al.). In another example, U.S.-based research institutions were challenged to address the minimal contributions of community voices and perspective within their research policies and practices (Eder). Further, findings from engaging “champion teams” from three very different academic health centers were presented as a way to improve organizational policies and practices to support equity based CBPR/CEnR (Sanchez-Youngman et al.). Another project involved developing a Principles of Trustworthiness toolkit to support how academic medical centers respond to and demonstrate to community partners that they are deserving of the community's trust as they work to advance health and social justice (Chinekezi et al.).

Studies in this Research Topic identify ways that community-engaged research can improve our understanding of individual and community outcomes and our ability to achieve them. In addition to structural barriers to engaging Oregon's Hispanic and Latino community members in cancer early detection research, the authors learned there was a low-level of community awareness of early detection cancer research, uncertainty about the benefits of research participation and few real opportunities for research participation within the community (Currier et al.). In study of a South African community, authors employed a modified random-route procedure to administer a standardized questionnaire in order to assess the extent to which community members were informed, consulted, involved and empowered to participate in two local public health projects (Mthembu and Chimbari). The authors concluded that while community members were largely educated, involved and informed, the projects lacked development of intrapersonal and personal project components that would better position community members to benefit. A longitudinal cohort study involving Native Hawaiian and Pacific Islanders created opportunities for cultural sharing and education, supporting generational change around diabetes (Mau et al.). Authors considered how deliberative democracy activities could successfully engage citizens of African Ancestry around the pros and cons of targeted screening for breast and ovarian cancer (Guan et al.). The approach resulted in sharing diverse and well-informed views, potentially avoiding misinformation. We also read about the testing of a Native-based nutrition and hypertension action oriented project, created using a participatory process with engaged American Indians and Alaska Natives (Sinclair et al.).

In the spirit of asserting agency and self-reflection within the cultural practices (15) that facilitated the development of this Research Topic, the editorial team identified challenges to the production of knowledge through peer review and publication. We were challenged by the process for identifying peers qualified to review each manuscript, beginning with the effort needed to identify and recruit qualified volunteer reviewers. This challenge may potentially be attributed to institutional expectations about faculty and scholars' responsibilities and priorities. We note with disappointment the withdrawal of a few manuscripts, perhaps due to irreconcilable cultural differences between local beliefs, practices and epistemological perspectives that diverge from a global tradition involving progress, imperialism, specialization and hierarchy. The review process may itself be critiqued as an academically driven process for exercising power over knowledge through the encouragement to correction and conformity with discursive structures that is both a model of and a model for a hegemonic epistemology (16–18). We acknowledge that the absent studies and their stories impeded the inclusion of diverse perspectives on knowledge production and the sharing of stories that diminishes science.

We also acknowledge that no commentaries were submitted that explored traditional measures of academic success such as securing grant funding or publication. These key metrics for community engaged scholarship now more commonly include expectations of joint academic and community member involvement. Common measures of scholarly accomplishment in combination with the scientific understandings of expertise and objectivity seem to be in tension with the goals of changing community-engaged health improvement research practices and policies, particularly with respect to the local dissemination and analysis of findings, to sustaining successful projects and partnerships, and policy development. It appears that current metrics of scholarly accomplishment represent biases that indicate misalignment with community-engaged, community-involved, community-based and participatory research practices. We further suggest that these metrics impede community members' willingness to engage and participate in (clinical) research and ultimately benefit from science (19).

At a fundamental level, all science requires self-reflection. We have considered how community-engaged and participatory research benefits from the deep knowledge, lived experience, and expertise of community members (15). In order for middle range theories to help community-engaged and participatory research teams navigate the theory to practice divide, we must understand both the relative consistency of biophysical interactions and the relative distributions of shared expectations and shared meanings among those involved. As community members increase their involvement in all aspects of the research enterprise and as they focus on addressing and improving community health, additional attention and self-reflection are needed to examine and explore the intersubjectivities encountered by individuals within communities who are both researchers and research participants. For now, the diversity of approaches in this Research Topic offers us an opportunity to celebrate different ways of knowing achieved through community research partnerships.

## Author contributions

ME initiated the Research Topic and took the lead in developing this introduction. KD'A, MY, and JO all edited the materials included in this Research Topic. All authors contributed to the article and approved the submitted version.
